# Magnesium sulfate and ophthalmic artery Doppler velocimetry in patients with severe preeclampsia: a case series

**DOI:** 10.1186/s13256-017-1490-1

**Published:** 2017-11-20

**Authors:** Cristiane Alves Oliveira, Renato Augusto Moreira de Sa, Karina Vieira Zamprogno, Fabio Gutierrez da Matta, Flávia do Vale Araújo

**Affiliations:** 1 Universidade Federal Fluminense - UFF, Niteroi, RJ Brazil; 2 Instituto Fernandes Figueira/Fiocruz, Rio de Janeiro, RJ Brazil; 3 Grupo Perinatal, Rio de Janeiro, RJ Brazil; 4 Rua Mário Alves 69/1204 Icaraí, Niteroi, RJ Brazil

**Keywords:** Hypertension, Ophthalmic artery, Ultrasonography, Doppler

## Abstract

**Background:**

In the present study, we used Doppler velocimetry in the ophthalmic artery to evaluate the hemodynamic status of the intracranial vasculature. This is the first time in the literature that indices of ophthalmic artery Doppler sonography of women with preeclampsia were evaluated before and after the use of magnesium sulfate to prevent eclampsia.

**Case presentation:**

Indices of ophthalmic artery Doppler sonography of six women with severe preeclampsia at 27 to 33 weeks of gestational age were evaluated before and after the use of magnesium sulfate (10 minutes, 30 minutes, and 60 minutes after the magnesium sulfate loading dosage. The patients’ ages were 26 years (patient 01), 29 years (patient 02), 20 years (patient 03), 21 years (patient 04), 20 years (patient 05), and 19 years (patient 06). The ethnic group of patients 01 and 04 was white and the ethnic group of patients 02, 03, 05 and 06 was mulatto.

**Conclusions:**

The apparent increase in resistance index and pulsatility index values, although there is no statistical significance in this series of cases, and the decrease in peak ratio values after the administration of magnesium sulfate reflect an increase in the impedance to flow in the ophthalmic artery and consequently a reduction in cerebral perfusion after the use of magnesium sulfate. This may explain how magnesium sulfate protects women with severe preeclampsia against cerebral damage and prevents acute convulsions in these patients. We believe that this case series report may have a broader clinical impact across medicine because the mechanism of how magnesium sulfate can protect patients and prevent acute convulsions is controversial.

## Background

Preeclampsia (PE) is one of the most important causes of maternal death and the acute cerebral complications in women with PE are responsible for at least 75% of these deaths [1–8]. Although both vasospasm and cerebral overperfusion may be associated with eclamptic seizures, in most cases, the cerebral damage in women with PE is associated to cerebral overperfusion rather than ischemia [7, 9, 10].

Since the ability to prevent PE is limited, management of pregnant women with PE has focused on identifying signs and symptoms of PE severity, with close clinical and laboratory monitoring to recognize the disease process in its early stages to prevent its complications [1, 11].

In women with severe PE, the use of magnesium sulfate (MgSO_4_) is indicated for prevention and control of acute convulsions. Several randomized trials have compared the efficacy of MgSO_4_ with other anticonvulsants in women with eclampsia, and the rates of recurrent seizures and maternal death were significantly reduced with MgSO_4_ as compared with other anticonvulsants [4, 5]. However, the anticonvulsant mechanism of MgSO_4_ is not fully elucidated and the cerebral hemodynamic effect of MgSO_4_ in PE is still under investigation [7, 12]. The total peripheral resistance reduction properties of MgSO_4_, which counteract the vasospasm induced by vasoconstrictor substances, can act on most types of calcium channels in vascular smooth muscle on voltage, receptor, and leak-operated membrane channels and as such would be expected to decrease intracellular calcium. Low intracellular calcium would inactivate calmodulin-dependent myosin light chain kinase activity and decreased contraction, causing arterial relaxation that may subsequently decrease peripheral and cerebral vascular resistance, relieving vasospasm, and decreasing arterial blood pressure [13, 14].

Some authors have suggested that MgSO_4_ has a cerebral (and retinal) vasodilator effect in women with PE; however, if in most severe cases, women with PE have cerebral overperfusion rather than ischemia, the question is: How can MgSO_4_ protect these patients and prevent acute convulsions? [7]. Belfort *et al*. [8], 2008, investigated the cerebral hemodynamic effect of MgSO_4_ in 15 women with PE. Transcranial Doppler of the middle cerebral arteries (MCA) was performed before and after intravascular administration of MgSO_4_. They observed reduction in cerebral perfusion pressure in those patients with high baseline cerebral perfusion pressure.

Ophthalmic artery Doppler is a noninvasive examination used to study central territory vascular flow during pregnancy. During pregnancy it could be useful in the differential diagnosis of PE and chronic arterial hypertension, and in the identification of severe cases of PE [1–3, 11, 15].

This study aimed to evaluate the ophthalmic Doppler indices before and after intravenously administered MgSO_4_ in woman with singleton pregnancies complicated by severe PE.

## Case presentations

Indices of ophthalmic artery Doppler sonography of six women with severe PE at 27 to 33 weeks of gestational age (GA) were evaluated before and after the use of MgSO_4_ (10 minutes, 30 minutes, and 60 minutes) after the MgSO_4_ loading dosage (4 g intravenously administered over 10 minutes), during the use of the MgSO_4_ maintenance dosage (1 g/hour intravenously administered), in a prospective observational study. All patients received antihypertensive therapy with hydralazine (5 mg intravenously administered over 2 minutes). The patients’ ages were 26 years (patient 01), 29 years (patient 02), 20 years (patient 03), 21 years (patient 04), 20 years (patient 05), and 19 years (patient 06). The ethnic group of patients 01 and 04 was white and the ethnic group of patients 02, 03, 05, and 06 is mulatto.

The definition of severe PE includes increased blood pressure (BP; systolic BP is 140 mmHg or a diastolic BP is 90 mmHg in a woman who was normotensive prior to 20 weeks of gestation) accompanied by proteinuria or increased BP accompanied by one or more severe complications even in the absence of proteinuria [4, 16]. The definition of severe PE was established in 2014 by the International Society for the Study of Hypertension in Pregnancy (ISSHP) [16]. The revised ISSHP definition of PE is hypertension developing after 20 weeks’ gestation and the coexistence of one or more of the following new onset conditions:

1. Proteinuria (0.3 g protein in a 24-hour specimen)
2. Other maternal organ dysfunction
  - Renal insufficiency (creatinine > 90 umol/L)
  - Liver involvement (elevated transaminases and/or severe right upper quadrant or epigastric pain)
  - Neurological complications (examples include eclampsia, altered mental status, blindness, stroke, or more commonly hyperreflexia when accompanied by clonus, severe headaches when accompanied by hyperreflexia, and persistent visual scotomata)
  - Hematological complications (thrombocytopenia, disseminated intravascular coagulation, hemolysis)
3. Uteroplacental dysfunction (fetal growth restriction)

Written informed consent was obtained from all participants. This study was authorized by the Research Ethics Committee of the Universidade Federal do Rio de Janeiro.

Maternal BP was measured prior to ocular Doppler sonography assessment, after a rest period of 10 minutes.

Scans were performed using Sonoace X8 equipment (high-resolution; Samsung Medison Co. Ltd, Seoul, South Korea) with a 7.5 MHz linear transducer, a 50 Hz wall filter setting, and the Doppler sample volume adjusted at 2 to 3 mm [1, 2, 11, 15, 17].

Patients were evaluated in the supine position and gel was applied to their closed right eyelid. After the ultrasound transducer was carefully placed on their eyelid, the blood flow waveform was obtained in their ophthalmic artery. The sample volume was oriented nasally and superior to the optic nerve, lateral to the hypoechoic stripe that represents it, 12 to 15 mm from the posterior wall of the sclera. The insonation angle was < 20 degrees. Six consecutive blood flow-velocity waveforms with similar size and shape were necessary before measurements were performed on a single waveform [1, 2].

The flow-velocity waveforms of the ophthalmic artery show a steep maximum systolic peak with a dicrotic protodiastolic notch and a low diastolic flow velocity. The resistance index (RI), the pulsatility index (PI), and the peak ratio (PR) were measured. The PR was defined as the ratio of the flow-velocity of the second peak (after the notch) to that of the initial peak (peak systolic velocity): PR = P2/P1 [1, 2].

The GA was calculated using the date of last menses, and it was confirmed with an ultrasound scan prior to 15 weeks of gestation. The criterion of complete weeks, as established by the World Health Organization (WHO), was used.

The mean time required for the analysis of the ophthalmic Doppler was approximately 5 minutes.

Statistical analysis: RI, PI, and PR indices were analyzed by analysis of variance (ANOVA). *P* < 0.05 was regarded as significant. Values are presented as mean ± standard deviation (SD).

**Fig. 1:**
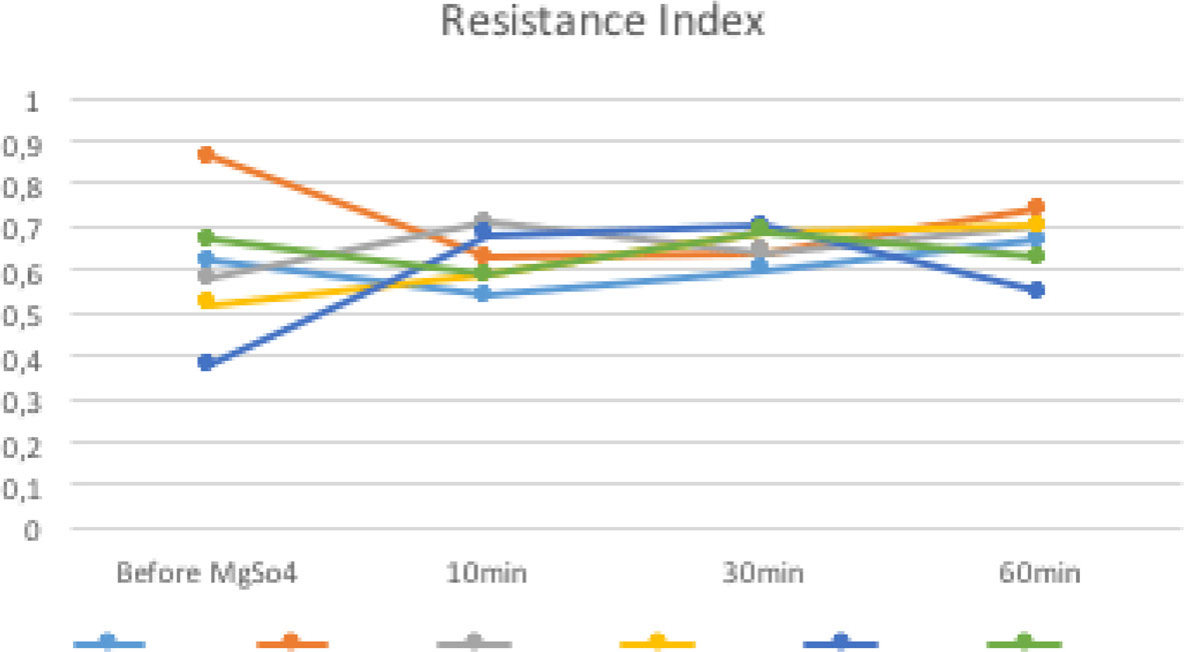
Resistance index before, 10 minutes, 30 minutes, and 60 minutes after the magnesium sulfate loading dosage for each patient. *MgSO*_*4*_ magnesium sulfate, *min* minutes

**Fig. 2:**
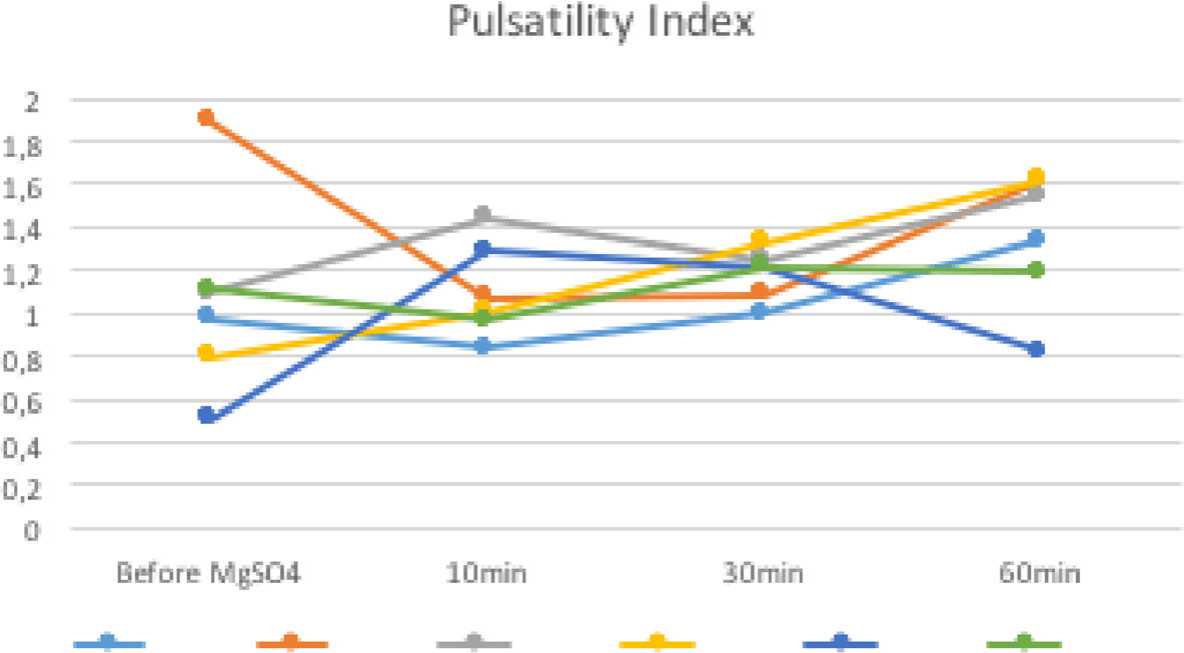
Pulsatility index before, 10 minutes, 30 minutes, and 60 minutes after the magnesium sulfate loading dosage for each patient. *MgSO*_*4*_ magnesium sulfate, *min* minutes

**Fig. 3:**
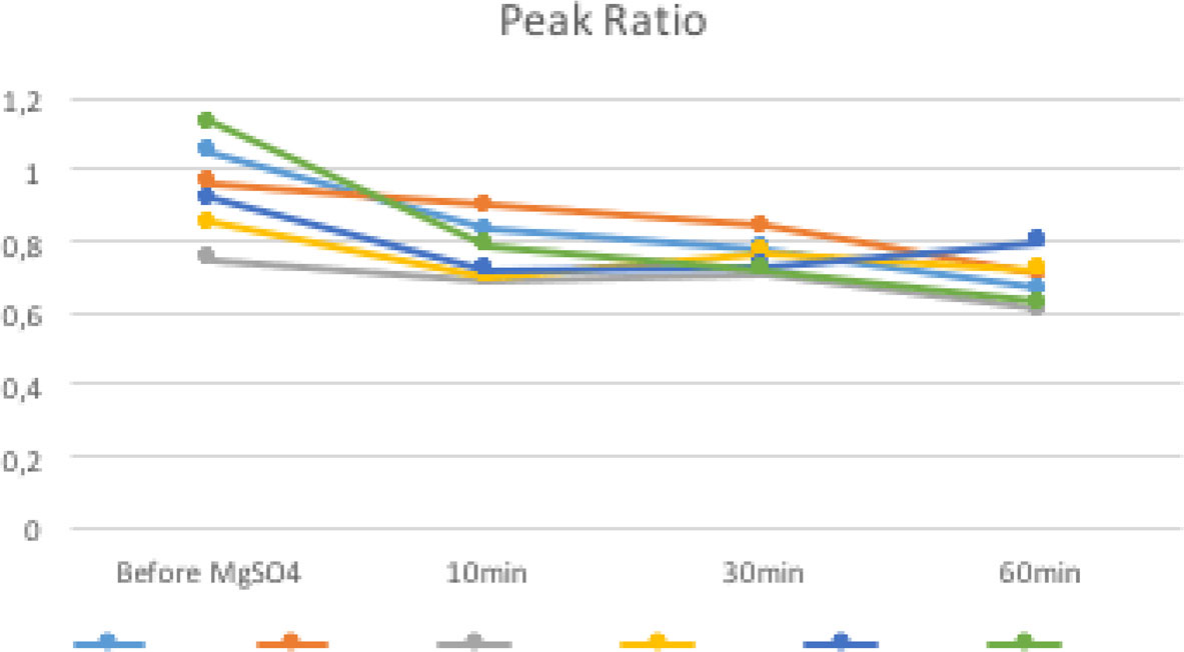
Peak ratio before, 10 minutes, 30 minutes, and 60 minutes after the magnesium sulfate loading dosage for each patient. *MgSO*_*4*_ magnesium sulfate, *min* minutes

All patients had systolic BP of 160 mmHg and diastolic BP of 110 mmHg. The mean values obtained for RI, before, 10 minutes, 30 minutes, and 60 minutes after the MgSO_4_ loading dosage were respectively: 0.605 (± 0.159 SD), 0.623 (± 0.063 SD), 0.660 (± 0.039 SD), and 0.665 (± 0.067 SD; Fig. 1). The mean values obtained for PI, before, 10 minutes, 30 minutes, and 60 minutes after the MgSO_4_ loading dosage were respectively: 1.065 (± 0.462 SD), 1.103 (± 0.221 SD), 1.185 (± 0.119 SD), and 1.355 (± 0.312 SD; Fig. 2). The mean values obtained for PR, before, 10 minutes, 30 minutes, and 60 minutes after the MgSO_4_ loading dosage were respectively: 0.943 (± 0.136 SD), 0.671 (± 0.091 SD), 0.758 (± 0.048 SD), and 0.690 (± 0.068 SD; Fig. 3).

The values obtained for RI, PI, and PR in the patients evaluated are shown in Table 1. The Doppler indices comparison was based on the comparison of before treatment indices with the last measurement 60 minutes after medication.

**Table 1:** Ophthalmic artery Doppler indices of six pregnant women with severe preeclampsia before the loading dosage of magnesium sulfate and after the use of magnesium sulfate (10, 30, and 60 minutes)

Patient	GA weeks	Maternal age (years)	RI				PI				PR			
			before MgSO_4_	10 min	30 min	60 min	before MgSO_4_	10 min	30 min	60 min	before MgSO_4_	10 min	30 min	60 min
01	27	26	0.62	0.54	0.60	0.67	0.98	0.84	1.00	1.34	1.05	0.83	0.78	0.67
02	29	29	0.86	0.63	0.64	0.74	1.89	1.07	1.09	1.61	0.96	0.90	0.84	0.71
03	31	20	0.58	0.71	0.64	0.70	1.10	1.44	1.25	1.55	0.75	0.69	0.71	0.61
04	33	21	0.52	0.59	0.69	0.70	0.80	1.01	1.33	1.62	0.85	0.70	0.77	0.72
05	31	20	0.38	0.68	0.70	0.55	0.51	1.29	1.22	0.82	0.92	0.72	0.73	0.80
06	30	19	0.67	0.59	0.69	0.63	1.11	0.97	1.22	1.19	1.13	0.79	0.72	0.63

*GA* gestational age, *MgSO*_*4*_ magnesium sulfate, *min* minutes, *PI* pulsatility index, *PR* peak ratio, *RI* resistance index

The PR indices were significantly lower in the 60 minutes after the MgSO_4_: *P* = 0.000192, F = 10.834 (one-way ANOVA). We observed no statistical difference in RI (*P* = 0.6445, F = 0.564; one-way ANOVA) and PI (*P* = 0.3872, F = 1.062; one-way ANOVA) after the MgSO_4_.

## Discussion

PE is a syndrome that is characterized by heterogeneous clinical and laboratory findings, and involves multiple organs (brain, liver, lungs, kidneys, and hematologic system). The clinical findings of PE can manifest as either a maternal syndrome, or a fetal syndrome (that is, fetal demise, fetal growth restriction), or both. The onset of seizures in pregnant women with PE defines a severe form of the disorder called eclampsia [1–5].

The etiology of eclamptic convulsions is unclear. Cerebral vasospasm, cerebral overperfusion, excitation of brain receptors, and a hyperactive sympathetic nervous system have been implicated as etiologic agents of eclampsia [7, 9, 10].

Over the years, the available clinical, pathological, and neuroimaging findings have led to two theories to explain the cerebral abnormalities associated with eclampsia: cerebral vasospasm and cerebral hyperperfusion. The first theory suggests that, in response to acute severe hypertension, cerebral “overregulation” leads to vasospasm. Vasospasm and diminished cerebral blood flow are hypothesized to result in cytotoxic edema, ischemia, and tissue infarction. The second theory suggests that sudden elevations in systemic BP may exceed the autoregulation of cerebral blood flow, a mechanism that aims to maintain adequate brain perfusion in abnormal situations, such as hypertension. Impaired cerebral autoregulation leads to forced vasodilatation, especially in the arterial boundary zones, with extravasation of plasma and red cells through opening of the endothelial tight junctions leading to a breakthrough brain edema. This phenomenon has been described as posterior reversible leukoencephalopathy syndrome (PRES). PRES is now hypothesized to be the primary injury in eclampsia. The pathophysiologic mechanism of PRES remains under investigation; however, endothelial damage is recognized as a major feature in the pathophysiologic mechanism of PE-eclampsia and as a relevant risk factor for PRES [6, 15, 17, 18].

The management of PE has been focused on identifying women at higher risk of severe complications in order to prevent them [1, 11]. The use of MgSO_4_ is indicated for cerebral protection in women with severe PE. Since publication of the MAGPIE trial, which involved 10,110 women with PE in 175 hospitals in 33 countries, which showed a significant reduction in the rate of eclampsia in women with PE treated with MgSO_4_, there has been a worldwide increase in the use of MgSO_4_ for seizure prophylaxis in women with PE [19]. Various theories have been promulgated to explain the mechanism of action of MgSO_4_ in the prevention of seizure, including peripheral neuromuscular blockade, membrane stabilization, N-methyl-D-aspartate (NMDA) receptor blocking activity, cerebral vasodilation, and calcium channel blocking action [8].

The anticonvulsant mechanism of MgSO_4_ is not fully elucidated [7, 12]. Although some authors have suggested that MgSO_4_ has a cerebral vasodilator effect in women with PE, others studies observed reduction in cerebral perfusion pressure in those patients with high baseline cerebral perfusion pressure. As in most cases of severe PE, women with PE have cerebral overperfusion rather than ischemia, the reduction in cerebral perfusion after the use of MgSO_4_ should explain the cerebral protection of this medication in severe PE.

In the present study, we used Doppler velocimetry in ophthalmic artery to evaluate the hemodynamic status of the intracranial vasculature. Some studies have shown low orbital impedance flow patterns in cases of severe PE compared with mild ones, demonstrated by the elevation in ophthalmic artery PR and reduction in RI and PI. Although the mechanism of elevation of PR in severe PE remains unclear, the PR has been proposed to be the most sensitive indicator of vascular changes associated with central overperfusion in these patients [1, 3, 11, 15, 17, 18, 20–22]. The apparent increase in RI and PI values, although there is no statistical significance in this series of cases, and the decrease in PR values after the administration of MgSO_4_ observed in our study, reflect an increase in the impedance to flow in the ophthalmic artery and consequently a reduction in cerebral perfusion after the use of MgSO_4_. This can explain how MgSO_4_ protects women with severe PE against cerebral damage and prevents acute convulsions in these patients.

Our proposal for a future study, following this research line of ophthalmic artery Doppler as a clinical tool to analyze the effects of MgSO_4_ on cerebral circulation, takes into account the feasibility of this procedure as a factor that could determine the success of the therapy. However, it would be important to include a previous analysis of the cerebral parenchyma by MRI, but it is necessary to consider the difficulty of transporting and executing MRI in a patient with a severe clinical condition. It is also important to consider the ethical implications because performing MRI could delay the beginning of the treatment with MgSO_4_.

## Conclusions

The decrease in PR values and the apparent increase in RI and PI values after the administration of MgSO_4_ reflect an increase in the impedance to flow in the ophthalmic artery and consequently a reduction in cerebral perfusion after the use of MgSO_4_. This can explain how MgSO_4_ protects women with severe PE against cerebral damage and prevents acute convulsions in these patients.

## Abbreviations

ANOVA: Analysis of variance
BP: Blood pressure
GA: Gestational age
ISSHP: International Society for the Study of Hypertension in Pregnancy
MgSO_4_: Magnesium sulfate
PE: Preeclampsia
PI: Pulsatility index
PR: Peak ratio
PRES: Posterior reversible leukoencephalopathy syndrome
RI: Resistance index
SD: Standard deviation

## Authors’ information

Zamprogno KV, da-Matta FG, and Araújo FV are registrars in Medical Science Postgraduate Course, Federal Fluminense University – Niteroi, RJ, Brazil. Oliveira CA is professor of Medicine at Federal Fluminense University. Moreira-de-Sa RA is professor of Medicine at the Medical Science Postgraduate Course, Federal Fluminense University – Niteroi, RJ, Brazil and Fetal Medicine researcher from Fernandes Figueira Institute from FIOCRUZ.

## References

[1] de Oliveira CA, de Sá RA, Velarde LG, da Silva FC, do Vale FA, Netto HC. Changes in ophthalmic artery Doppler indices in hypertensive disorders during pregnancy. J Ultrasound Med. 2013;32:609–16. 10.7863/jum.2013.32.4.609 23525385

[2] de Oliveira CA, de Sá RA, Velarde LG, Marchiori E, Netto HC, Ville Y. Doppler velocimetry of the ophthalmic artery in normal pregnancy: reference values. J Ultrasound Med. 2009;28:563–9. 10.7863/jum.2009.28.5.563 19389894

[3] Matias DS, Costa RF, Matias BS, Correia LC. Doppler velocimetry of the orbital vessels in pregnancies complicated by preeclampsia. J Clin Ultrasound. 2012;40:576–85. 10.1002/jcu.21949 22729810

[4] Magee LA, Helewa ME, Moutquin J-M, von Dadelszen P. SOGC clinical practice guideline on Diagnosis, Evaluation, and Management of the hypertensive Disorders of Pregnancy. J Obstet Gynaecol Can. 2010;206:S1–48.

[5] Sibai B, Dekker G, Kupferminc M. Pre-eclampsia. Lancet. 2005;365:785–99. 10.1016/S0140-6736(05)17987-2 15733721

[6] Sibai BM. Hypertensive disorders of pregnancy: the United States perspective. Curr Opin Obstet Gynecol. 2008;20:102–6. 10.1097/GCO.0b013e3282f73380 18388806

[7] Belfort MA, Clark SL, Sibai B. Cerebral hemodynamics in preeclampsia: cerebral perfusion and the rationale for an alternative to magnesium sulfate. Obstet Gynecol Surv. 2006;61:655–65. 10.1097/01.ogx.0000238670.29492.84 16978425

[8] Belfort M, Allred J, Dildy G. Magnesium sulfate decreases cerebral perfusion pressure in preeclampsia. Hypertens Pregnancy. 2008;27:315–27. 10.1080/10641950801955683 19003633

[9] Ayaz T, Akansel G, Hayirlioglu A, Arslan A, Suer N, Kuru I. Ophthalmic artery color Doppler ultrasonography in mild-to-moderate preeclampsia. Eur J Radiol. 2003;46:244–9. 10.1016/s0720-048x(02)00194-8 12758119

[10] Ohno Y, Kawai M, Wakahara Y, Kitagawa T, Kakihara M, Arii Y. Ophthalmic artery velocimetry in normotensive and preeclamptic women with or without photophobia. Obstet Gynecol. 1999;94:361–3. 10.1016/s0029-7844(99)00294-x 10472860

[11] Diniz AL, Moron AF, dos Santos MC, Sass N, Pires CR, Debs CL. Ophthalmic artery Doppler as a measure of severe preeclampsia. Int J Gynaecol Obstet. 2008;100:216–20. 10.1016/j.ijgo.2007.07.013 17963761

[12] Souza AS, Amorim MM, Santos RE, Noronha Neto C, Porto AM. Effect of magnesium sulfate on pulsatility index of uterine, umbilical and fetal middle cerebral arteries according to the persistence of bilateral diastolic notch of uterine arteries in patients with severe preeclampsia. Rev Bras Ginecol Obstet. 2009;31:82–8. 10.1590/s0100-72032009000200006 19407913

[13] Kemp PA, Gardiner SM, March JE, Rubin PC, Bennett T. Assessment of the effects of endothelin-1 and magnesium sulphate on regional blood flows in conscious rats, by the coloured microsphere reference technique. Br J Pharmacol. 1999;126(3):621–6. 10.1038/sj.bjp.0702342 PMC1565849 10188972

[14] Korish AA. Magnesium sulfate therapy of preeclampsia: an old tool with new mechanism of action and prospect in management and prophylaxis. Hypertens Res. 2012;35(10):1005–11. 10.1038/hr.2012.103 22763474

[15] Carneiro RS, Sass N, Diniz AL, Souza EV, Torloni MR, Moron AF. Ophthalmic artery Doppler velocimetry in healthy pregnancy. Int J Gynaecol Obstet. 2008;100:211–5. 10.1016/j.ijgo.2007.09.028 18045602

[16] Tranquilli AL, Dekker G, Magee L, Roberts J, Sibai BM, Steyn W, Zeeman GG, Brown MA. The classification, diagnosis and management of the hypertensive disorders of pregnancy: A revised statement from the ISSHP. Pregnancy Hypertens. 2014;4:97–104. 10.1016/j.preghy.2014.02.001 26104417

[17] Barbosa AS, Pereira AK, Reis ZSN, Lage EM, Leite HV, Cabral ACV. Ophthalmic Artery-Resistive Index and Evidence of Overperfusion-Related Encephalopathy in Severe Preeclampsia. Hypertension. 2010;55:189–93. 10.1161/HYPERTENSIONAHA.109.143586 19948984

[18] Zeeman GG. Neurologic complications of pre-eclampsia. Semin Perinatol. 2009;33:166–72. 10.1053/j.semperi.2009.02.003 19464507

[19] The Magpie Trial Collaboration Group. Do women with preeclampsia, and their babies, benefit from magnesium sulphate? The Magpie Trial: a randomized placebo-controlled trial. Lancet. 2002;359:1877–90. 10.1016/s0140-6736(02)08778-0 12057549

[20] Nakatsuka M, Takata M, Tada K, Kudo T. Effect of a nitric oxide donor on the ophthalmic artery flow velocity waveform in preeclamptic women. J Ultrasound Med. 2002;21:309–13. 10.7863/jum.2002.21.3.309 11883542

[21] Barbosa AS. Estudo da Associação entre as Manifestações Oftálmicas da Pré-eclâmpsia Grave e os Parâmetros de Fluxo Sanguíneo das Artérias Oftálmica e Central da Retina ao Ecodoppler Ocular. Belo Horizonte: Tese (Doutorado), Universidade Federal de Minas Gerais; 2004.

[22] Hata T, Hata K, Moritake K. Maternal ophthalmic artery Doppler velocimetry in normotensive pregnancies and pregnancies complicated by hypertensive disorders. Am J Obstet Gynecol Surv. 1997;177:174–8. 10.1016/s0002-9378(97)70458-7 9240603

